# Role of the PD-1/PD-L1 Dyad in the Maintenance of Pancreatic Immune Tolerance for Prevention of Type 1 Diabetes

**DOI:** 10.3389/fendo.2020.00569

**Published:** 2020-08-19

**Authors:** Marika Falcone, Georgia Fousteri

**Affiliations:** Division of Immunology, Transplantation, and Infectious Diseases, San Raffaele Scientific Institute, DRI - Diabetes Research Institute, Regulation of Adaptive Immunology, Milan, Italy

**Keywords:** type 1 diabetes (T1D), programmed death 1 (PD-1), programmed death ligand 1 (PD-L1), immune tolerance, immune homeostasis, immunotherapy, pancreatic islet transplantation, pancreas

## Abstract

The human pancreas, like almost all organs in the human body, is immunologically tolerated despite the presence of innate and adaptive immune cells that promptly mediate protective immune responses against pathogens *in situ*. The PD-1/PD-L1 inhibitory pathway seems to play a key role in the maintenance of immune tolerance systemically and within the pancreatic tissue. Tissue resident memory T cells (TRM), T regulatory cells (Treg), macrophages and even β cells exhibit PD-1 or PD-L1 expression that contributes in controlling pancreatic immune homeostasis and tolerance. Dysregulation of the PD-1/PD-L1 axis as shown by animal studies and our recent experience with checkpoint inhibitory blockade in humans can lead to immune dysfunctions leading to chronic inflammatory disease and to type 1 diabetes (T1D) in genetically susceptible individuals. In this review, we discuss the role of the PD-1/PD-L1 axis in pancreatic tissue homeostasis and tolerance, speculate how genetic and environmental factors can regulate the PD-1/PD-L1 pathway, and discuss PD-1/PD-L1-based therapeutic approaches for pancreatic islet transplantation and T1D treatment.

## Introduction

Type 1 diabetes (T1D) is an autoimmune disease mediated by T-cell destruction of the insulin-producing β-cells in the pancreatic islets of Langerhans (1). The critical link between the Programmed death 1 (PD-1)/PD-L1 pathway and constraint of T1D has been demonstrated in numerous studies and has paved the way for novel therapeutic approaches. PD-1 is an inhibitory molecule belonging to the class of co-stimulatory molecules expressed on the surface of T cells that has been linked to immune tolerance (2). PD-1 is a member of the CD28 and CTLA-4 immunoglobulin superfamily and interacts with two B7 family ligands, PD-L1 (CD274) and PD-L2 (CD273) (3). PD-L1 is widely distributed on leukocytes and non-hematopoietic cells in lymphoid and non-lymphoid tissues, including pancreatic islets, whereas PD-L2 is expressed exclusively on dendritic cells (DCs) and monocytes (4, 5).

Upon binding to ligands PD-L1 and PD-L2, PD-1 recruits SHP2 phosphatase, which then dephosphorylates molecules downstream of the TCR and CD28, leading to a block in T cell effector function (6). Thus, PD-1 blockade can reinvigorate exhausted T cells, providing enhanced antiviral and antitumor responses (7, 8). These observations have led to the development of PD-1 immune checkpoint inhibitors (ICI), which have revolutionized cancer therapy (9). Interestingly, adverse events such as rapid autoimmunity including T1D developed following checkpoint blockade in cancer patients (10–13), possibly due to reversal of T cell exhaustion in pancreatic islets (14). Those findings suggested a key role for the PD1-PD-L1 inhibitory pathway in the maintenance of immune homeostasis and tolerance in pancreatic tissue and the prevention of T1D. Here, we discuss the role of PD-1 in pancreas immune homeostasis and tolerance and the progress made so far in exploiting the PD-1/PD-L1 dyad as a means to prevent and/or treat T1D.

## The PD-1/PD-L1 Axis Promotes Beta Cell Tolerance and Prevents T1D

Several lines of evidence indicate that the PD-1/PD-L1 axis is fundamental to maintain immune homeostasis and prevent organ-specific autoimmune diseases such as T1D. The importance of this inhibitory pathway in the pathophysiology of T1D has been demonstrated in mice and humans. The Non-obese Diabetic (NOD) mice develop spontaneous T1D and represent the most used murine pre-clinical models of T1D. For example, NOD mice deficient for PD-1 or PD-L1 develop accelerated T1D (15, 16). Using insulin tetramers, Pauken et al., quantified insulin-specific CD4 T cells in the secondary lymphoid organs (SLO) and pancreas of NOD.PD-1^−/−^ mice (17). They observed significantly more insulin-specific T cells in the pancreatic LN (pancLN) of prediabetic and diabetic NOD.PD-1^−/−^mice compared to WT NOD controls. Furthermore, the same group observed that selective loss of PD-1 on islet-reactive CD4 T cells enhanced their proliferation and recruitment in pancreatic islets (18). Antibody blockade experiments showed that PD-1:PD-L1 interactions, but not PD-1:PD-L2, were necessary for the maintenance of tolerance toward pancreatic islets in the NOD mice (19–25). Interestingly, genetic deletion of PD-1 in C57BL/6 and BALB/c mice led to spontaneous lupus-like disease or autoimmune cardiomyopathy, respectively, but no T1D (26, 27), thus implying that a defective PD-1/PD-L1 inhibitory pathway is not sufficient to trigger autoimmune diabetes. Even in B6.g7 mice sharing the MHCII with NOD mice and carrying high genetic risk for T1D, treatment with anti-PD-1 was not enough to induce T1D (17).

In humans, a possible role for the PD-1/PD-L1 axis in T1D pathogenesis is suggested by the observation that recent onset T1D patients have elevated gene expression levels of CD274 (PD-L1) in whole-blood RNA analysis (25). In addition, both decreased PD-1 gene expression in peripheral CD4^+^ T cells and low frequency of circulating PD-1^+^ CD4^+^ T cells were found in T1D patients (25, 28). More recently, Granados et al., demonstrated that peripheral T cells from children with new onset T1D failed to upregulate PD-1 upon T-cell receptor stimulation (29). Also, the CD4^+^ CD25^+^ Treg cells of T1D patients are defective in their ability to upregulate PD-1 and to efficiently use the PD-1/PD-L pathway to mediate their immunosuppressive function (30).

The importance of the PD-1/PD-L1 pathway in maintenance of immune tolerance toward pancreatic beta cells in humans is furtherly highlighted by the observation that 0.4–2.0% of individuals undergoing treatment with ICI (anti-PD-1 and/or anti-PD-L1 mAb) develop T1D (11–13). In a recent review of the literature, 90 clinical cases of T1D induced by ICI were reported (14). In 51% of cases, T1D onset was associated with occurrence of one or more autoantibodies against islet antigens. Genotype associated with T1D susceptibility were present in 61% of cancer patients who developed T1D upon ICI treatment (11–13). These findings indicate that the PD-1/PD-L1 axis plays a key role in maintenance of immune homeostasis and tolerance to pancreatic antigens. T1D is a multifaceted disease regulated by genetic and environmental factors whose pathogenesis could be very diverse in different T1D patients. In fact, in individuals diagnosed with T1D sharing common clinical signs of the disease, the triggering pathogenic events leading to autoimmune destruction of pancreatic islets maybe very different. Hence, a defect of the PD-1/PD-L1 dyad could lead to T1D in a subgroup of patients as the anti-PD-1/PD-L1 therapy triggers T1D in a percentage of individuals and, particularly, on those who carry other T1D susceptibility genes.

How does the PD-1/PD-L1 axis control β cell autoimmunity? Expression of PD-1 on T cells controls their activation and drives them toward exhaustion. T-cell exhaustion is an important mechanism to maintain immune homeostasis and prevent autoimmune diseases including T1D (7). In support to this idea, a recent study demonstrated that slow T1D progression was associated with an exhaustion-like profile on islet-reactive T cells, with expression of multiple inhibitory receptors (including PD-1), limited cytokine production, and reduced proliferative capacity (31). Along the same line, an increase in circulating exhausted T cells predicted response to anti-CD3 therapy in T1D (32). FcR-non-binding anti-CD3 mAb immunotherapy is effective in delaying T1D occurrence in subjects with risk to develop the disease (autoantibody-positive) (33–35). Importantly, Fife et al., identified a critical role for PD-1/PD-L1 in the response of T1D patients to anti-CD3 immunotherapy (22), suggesting that PD-1–PD-L1 interactions are part of a common pathway to selectively maintain tolerance within the pancreatic tissue and the draining lymph nodes possibly through induction of T cell exhaustion.

Antigen-specific therapy is another highly promising therapeutic approach to harness the progression of T1D (36–38) that could also exploit the PD-1-PD-L1 inhibitory pathway. Using this approach, we and other groups have demonstrated disease remission, inhibition of pathogenic T cell proliferation and anergy, decreased pro-inflammatory cytokine production, and regulatory cytokine and T cell induction (39–44). Fife et al., showed that an antigen-specific therapy with insulin-coupled antigen-presenting cells was able to revert T1D in NOD mice after disease onset (22). Importantly, robust long-term tolerance following this treatment was dependent on the PD-1–PD-L1 pathway (22). Anti–PD-1 and anti–PD-L1, but not anti–PD-L2, reversed tolerance weeks after tolerogenic therapy by promoting antigen-specific T cell proliferation and inflammatory cytokine production directly in infiltrated tissues (22), thus suggesting that the PD-1/PD-L1 blockade at pancreatic tissue level maybe important. Following a similar approach, administration of the islet antigen peptide mimic p31 coupled to chemically fixed antigen presenting cells (APCs) reversed diabetes and induced robust, long-term inactivation of islet-specific BDC2.5 T-cell receptor (TCR)–transgenic T cells (23). Here, both PD-1 and CTLA-4 interactions were critical for the induction of tolerance. However, long-term maintenance of the anergic T cell state exclusively depended on PD-1/PD-L1 pathway (23). Additional experiments indicated that PD-1 acted in a cell-intrinsic manner to maintain tolerance.

One hallmark of T1D is the presence of islet-specific autoantibodies (45, 46) whose production depends on cognate interactions between a specialized subset of CD4 T cells known as T follicular helper (Tfh) and B cells in the germinal centers (GC) (47, 48). Tfh cells express PD-1, ICOS, CXCR5, and Bcl-6 and provide IL-4, IL-21, and CD40-ligand stimulation to developing/maturing B cells, thus promoting antibody affinity maturation and somatic hypermutation (49, 50). Increases in the number of circulating Tfh cells, and, importantly, elevated expression of an activation phenotype i.e., elevation of ICOS and PD-1 expression, have been reported in patients with autoimmunity including T1D, suggesting that these cells may contribute to disease development (51–57). T follicular regulatory (Tfr) cells are a subset of FOXP3 Treg cells that also express PD-1, ICOS, CXCR5, CD25, Bcl-6, and Foxp3 and suppress Tfh–B cell interactions to limit autoimmunity (58, 59). In children with new-onset T1D, a reduction of PD-1 expression on Tfr cells was observed in a recent study (60). Additionally, children with T1D and dysregulated PD-1 expression were shown to be more susceptible to autoimmune complications of T1D, such as celiac disease and thyroiditis (29). These studies highlight that the PD-1 and PD-L1 axis plays an important role in regulating CD4 T cell–B cell crosstalk, the development of autoantibodies and the severity of T1D.

In recent work, PD-1 blockade was shown to enhance both the Tfh and Tfr CD4 T cells, but their ratio determined the final outcome of the GC response during foreign antigen immunization and in experimental autoimmune encephalomyelitis (61). In the NOD mouse model of T1D, Martinov et al., demonstrated that PD-1 or PD-L1 deficiency, as well as PD-1 but not PD-L2 blockade, increased both insulin-specific Tfh and Tfr cells and increased their survival (61). Additionally, PD-1 deficiency resulted in an increase in insulin-specific B cells and insulin autoantibodies (IAAs) in the mouse sera (61). The increase in insulin-specific Tfh/Tfr cell ratio after PD-1 blockade possibly accounted for the increased IAA production, similarly to what has been described previously for bulk Tfh/Tfr cell ratio (59). Interestingly, using an antibody that specifically disrupts TCR interactions with insulin peptide:MHC II complex, reduced the effects of PD-1 blockade on insulin-reactive B cell expansion but did not impact T1D incidence (61).

## The PD-1/PD-1L Pathway is Fundamental To Maintain Immune Homeostasis In The Pancreatic Tissue

The PD-1/PD-L1 axis is instrumental for maintenance of immune homeostasis in several organs including the pancreatic tissue as suggested by the observation that blockade of the PD-1/PD-L1 pathway in 1.8% of cancer patients treated with anti-PD-1 antibodies results in acute or chronic pancreatitis (62). Furthermore, several lines of evidence indicate that this inhibitory pathway is particularly important to maintain immune tolerance against insulin-producing pancreatic β cells for prevention of T1D. Pancreatic β cells express very low levels of PD-L1 in basal conditions, however inflammation triggers higher expression mostly through the action of cytokines such interferons (63, 64). Osum et al., found that IFN-γ and, to a lesser extent, IFN-α, promoted increased frequency of PD-L1+ β cells, and increased expression of PD-L1 on a per cell basis (63). The fact that PD-L1 expression is upregulated in inflamed islets and, specifically, in the presence of CD8+ T-cell infiltration suggests that this could represent a key mechanism to control T cell activation and promote T cell exhaustion in pancreatic tissues (63).

CD8 T cells, most likely islet-specific, are found within islets and the insulitic lesions as well as in the exocrine pancreas of T1D patients (5, 16, 65–69), hence the PD-1/PD-L1-mediated control of CD8 T cell infiltration may play an important role in prevention of T1D. In support to this hypothesis, autoantibody positive patients without clinically overt T1D showed a slight increase in PD-L1 expression on residual pancreatic islets, thus suggesting that PD-L1 expression maybe protective (63). Along the same line, PD-L1 expression was absent from insulin-deficient islets where β cells had been destroyed by the autoimmune process (63).

The mechanism through which the PD-1 inhibitory pathway regulates T1D development within pancreatic tissues was elegantly addressed *in vivo* in pre-clinical models of T1D by multiphoton imaging techniques. Those experiments showed that PD-1 suppressed TCR-driven stop signals in the pancreatic islets. Moreover, they showed that blockade of PD-1 or PD-L1 inhibited T cell migration, prolonged T cell–DC engagement, enhanced T cell cytokine production, boosted TCR signaling and abrogated peripheral tolerance (23).

**Figure 1:**
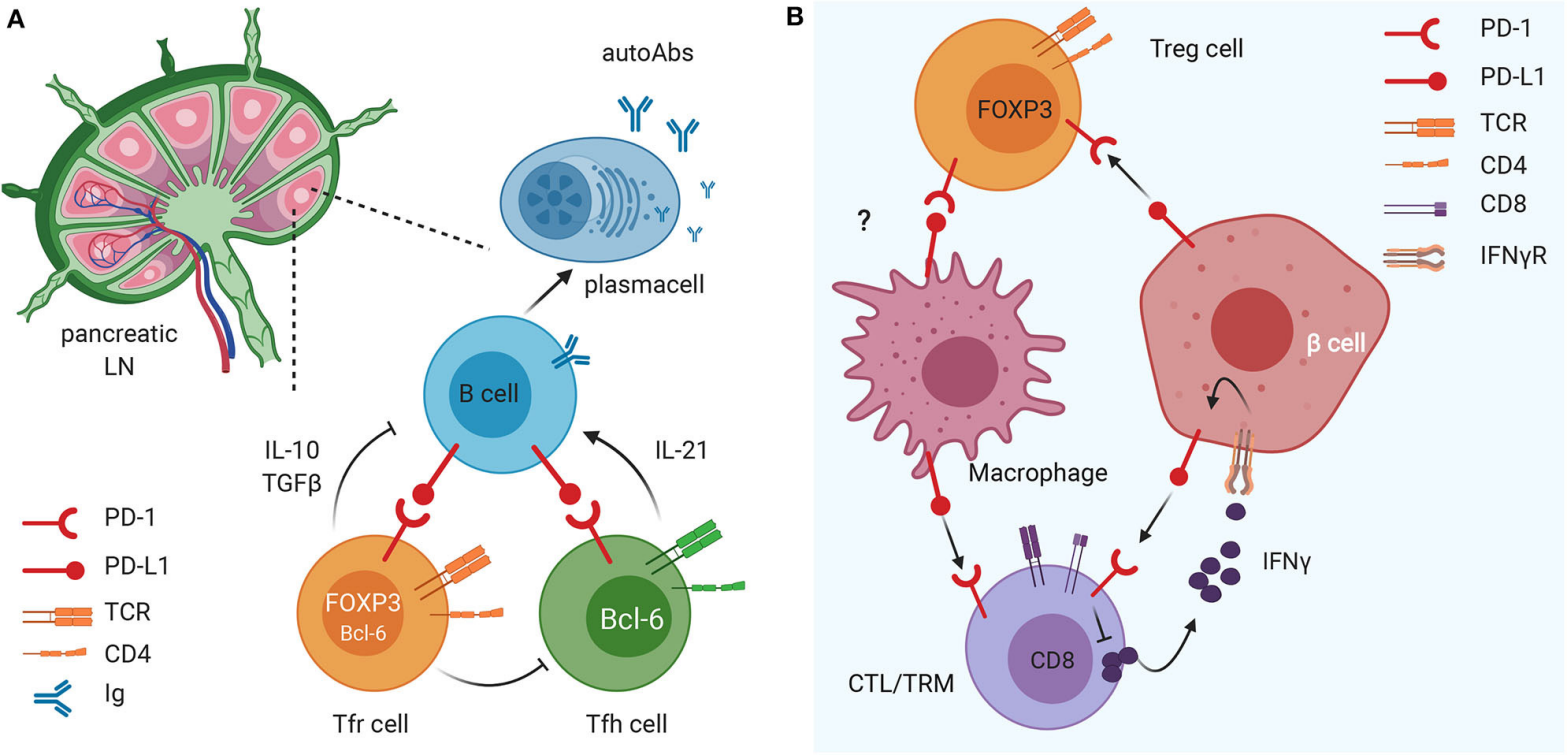
The immunomodulatory role of the PD1/PD-L1 axis on different innate and adaptive immune cell subsets in pancreatic lymph nodes (LN) and tissue. **(A)** The PD1/PD-L1 dyad is crucial in the crosstalk between B cells and Tfollicular helper (Tfh) and follicular regulatory (Tfr) cells in the draining LN. This mechanism favors maturation of B cells and their release of islet-specific autoAbs and could have important implications in T1D pathogenesis. **(B)** PD-L1 expression on insulin-secreting 13 cells of the pancreatic islets of Langerhans down-regulates the activation and promotes exhaustion of autoreactive CD8 T cells and enhances suppressive function of FoxP3+ Treg cells. In parallel, PD-L1 expression on tissue resident memory (TRM) cells (and, possibly, Treg cells) regulates macrophage recruitment and function within pancreatic tissues.

Recent studies indicated that the PD-1/PD-L1 dyad could be important in regulating activation of tissue-resident memory T cells (TRMs), a subset of T cells residing in the pancreatic tissue under steady-state conditions (70–72), that play a key role in pancreas immune surveillance and immunopathology in health and disease (66, 67, 73, 74). TRM cells exhibit site-specific functional and transcriptional adaptations in certain tissues including the pancreas (75, 76), playing an important role in mediating tissue homeostasis. Functionally, TRM cells rapidly release interleukin-2 (IL-2) and pro-inflammatory cytokines to mediate immediate protective responses against multiple types of pathogens and they can also participate in tissue immunopathology (77–79). Importantly, TRM cells normally express molecules that attenuate their activation such as the inhibitory molecules PD-1 and CD103 and the regulatory cytokine IL-10 (80, 81). In the human healthy pancreas TRM cells express elevated PD-1 levels compared to the intestinal mucosa (jejunum) and pancreatic draining lymph nodes (82), thus suggesting that their activation is tightly regulated by the PD-1/PD-L1 pathway. Pancreas TRM cells exhibited tissue-specific phenotypes and transcriptional programs controlling T cell activation and metabolism (82). All together these findings indicate that in steady-state conditions, PD-1/PD-L1 triggering on TRM cells is fundamental to halt their activation and maintain immune homeostasis within pancreatic tissues. Polyclonal TRM cells are present in T1D patients, particularly in the exocrine pancreas, but their exact role in T1D development is unknown. Another major immune cell type in the exocrine pancreas are macrophages that enhance TRM cells' functions (82). Interestingly, in studies conducted on pancreatic tissues of patients with chronic pancreatitis, TRM cells exhibited reduced PD-1 expression concomitant with a marked decrease in pancreas macrophages (82). Together, these findings suggest that TRM cells, macrophages, and the PD-1 pathway contribute to *in situ* immune regulation in the pancreas and a dysregulation of this immune regulatory pathway could contribute to the pathogenesis of T1D (Figure 1). Multiple factors could lead to breakage of central or peripheral immune tolerance and onset of β cell autoimmunity in T1D susceptible individuals. The expression of PD-1/PD-L1 molecules on β cells and tissue-resident immune cells could represent the ultimate safety mechanism to prevent autoimmune destruction of the pancreatic islets in individuals with β cell autoimmunity whose islet-reactive T cells are activated and recruited within the pancreatic tissue. Hence, in some individuals with high genetic risk of T1D and β cell autoimmunity, a dysregulation of the PD-1/PD-L1 inhibitory pathway could be an additional mechanism leading to T1D.

## Genetic and Environmental Factors Controlling The PD-1/PD-L1 Pathway

The aforementioned studies suggest that the PD-1/PD-L1 pathway maybe fundamental to control immune activation of islet-reactive T cells and TRM cells and to maintain immune homeostasis and tolerance in the pancreas. The onset of T1D induced in some individuals treated with ICI (i.e., anti-PD-1 mAb) indicates that a failure of these control mechanisms could be one of the mechanisms leading to β cell autoimmunity also in patients with “classical” T1D. Altered PD-1 expression on islet-reactive T cells and/or polyclonal TRM cells as well as defective PD-L1 expression on pancreatic islets could lead to failure of PD-1/PD-L1-mediated tolerance and immune homeostasis, ultimately leading to T1D. How is the PD-1-PD-L1 pathway regulated and how it contributes to T1D development? Both genetic and environmental factors modulate the PD-1/PD-L1 pathway and maybe involved in its dysregulation in T1D. Polymorphisms of the PD-1 gene (*PDCD1*) have been found in different autoimmune diseases and confers genetic susceptibility also to T1D (83). In humans, several studies have been performed to assess the effects of *PD-1* gene polymorphisms on T1D (84) and few single nucleotide polymorphisms associated with T1D were identified such as rs2227981 (PD-1.5), rs2227982 (PD-1.9) (85). Importantly, a recent study demonstrated that rs2227982 had a significant association with clinical signs of T1D (i.e., hyperglycemia), thus suggesting that the *PD-1* gene polymorphisms participate in increasing T1D risk (85).

Recently, a key role for the microbiota in controlling the PD-1/PD-L1 pathway expression and function has been identified. This finding has important implication for disease prevention as diet, antibiotic assumption and others environmental factors could affect the PD-1/PD-L1 function indirectly by altering the microbiota profile. Specifically, it was demonstrated that primary resistance to anti-PD-1 immune-checkpoint immunotherapy (ICI) in cancer patients is related to abnormal gut microbiome composition (86). Importantly, transfer of gut microbiota (fecal material transfer) from ICI responders into ICI resistant patients, increased the response to the anti-PD-1 treatment indicating that the components of the microbial strains could directly or indirectly act on the PD-1/PD-L1 axis (87). Also, modification of the microbiota induced by antibiotic treatment reduced the response to ICI suggesting that antibiotics could affect the inhibitory PD-1/PD-L1 axis by acting on the microbiota (86).

The mechanism underlying microbiota-induced modulation of PD-1/PD-L1 was analyzed in a murine model. Strikingly, it was found that administration of the anti-PD-1 mAb unleashed activation and recruitment of central memory T cells (T_CM_) into draining lymph nodes and within the tumor and increased the Teff/Treg cell ratio but only if specific bacterial strains (*A. muciniphila* and *E. hirae*) were present in the intestine of the tumor-bearing mice (86). These bacterial species may restore gut barrier integrity and reduced bacterial translocation that could induce immunosuppression of anti-tumor immunity. Alternatively, some microbiota strains could regulate PD-1 expression on T cells and/or PD-L1 expression on tumor cells thus increasing the therapeutic response to anti-PD-1.

The interaction between the microbiota and the PD-1-PD-L1 pathway is bi-directional. In fact, important evidence exists that the PD-1-PD-L1 axis regulate the gut microbiota composition. Kawamoto et al., (88) demonstrated that PD-1^−/−^ mice have an altered microbiota profile. Importantly, they showed that PD-1 modulated the gut bacterial communities through selection of IgA plasmacell repertoires. PD-1 deficiency generated an excess number of Tfh cells with altered phenotypes resulting in dysregulated selection of IgA-secreting B cells in the GCs of Peyer's patches. The IgA produced in PD-1^−/−^ mice have reduced bacteria-binding capacity, which causes alterations of the gut microbiota composition.

Considering the important role of the gut microbiota in modulating T1D pathogenesis (89–93), it is possible to speculate that dysregulation of the PD-1/PD-L1 pathway could affect diabetogenesis also by modifying the microbiome profiles. On the other hand, since some commensal bacterial strains modulate the response to anti-PD-1 therapy, the alteration of microbiota composition found in T1D patients could be directly or indirectly responsible for a defect of the PD-1-PD-L1 inhibitory pathway leading to reduced islet-reactive T cell exhaustion, enhanced activation of TRM cells in pancreatic tissue and β cell damage. This process could be triggered by components of the gut commensal microbiota translocating from the intestine to the pancreatic tissue. However, recent evidence indicates that not only the gut microbiota but also organ-specific commensal strains, i.e., skin-resident bacteria, play immunoregulatory function modulating skin graft rejection (94). Hence, future studies are necessary to clarify whether tissue-resident microbiota also exists in pancreatic tissues and, importantly, whether they are involved in maintenance of immune homeostasis and tolerance toward β cells possibly through modulation of the inhibitory PD-1/PD-L1 pathway.

## How Could The PD-1/PD-1L Pathway Be Therapeutically Exploited In T1D?

**Figure 2:**
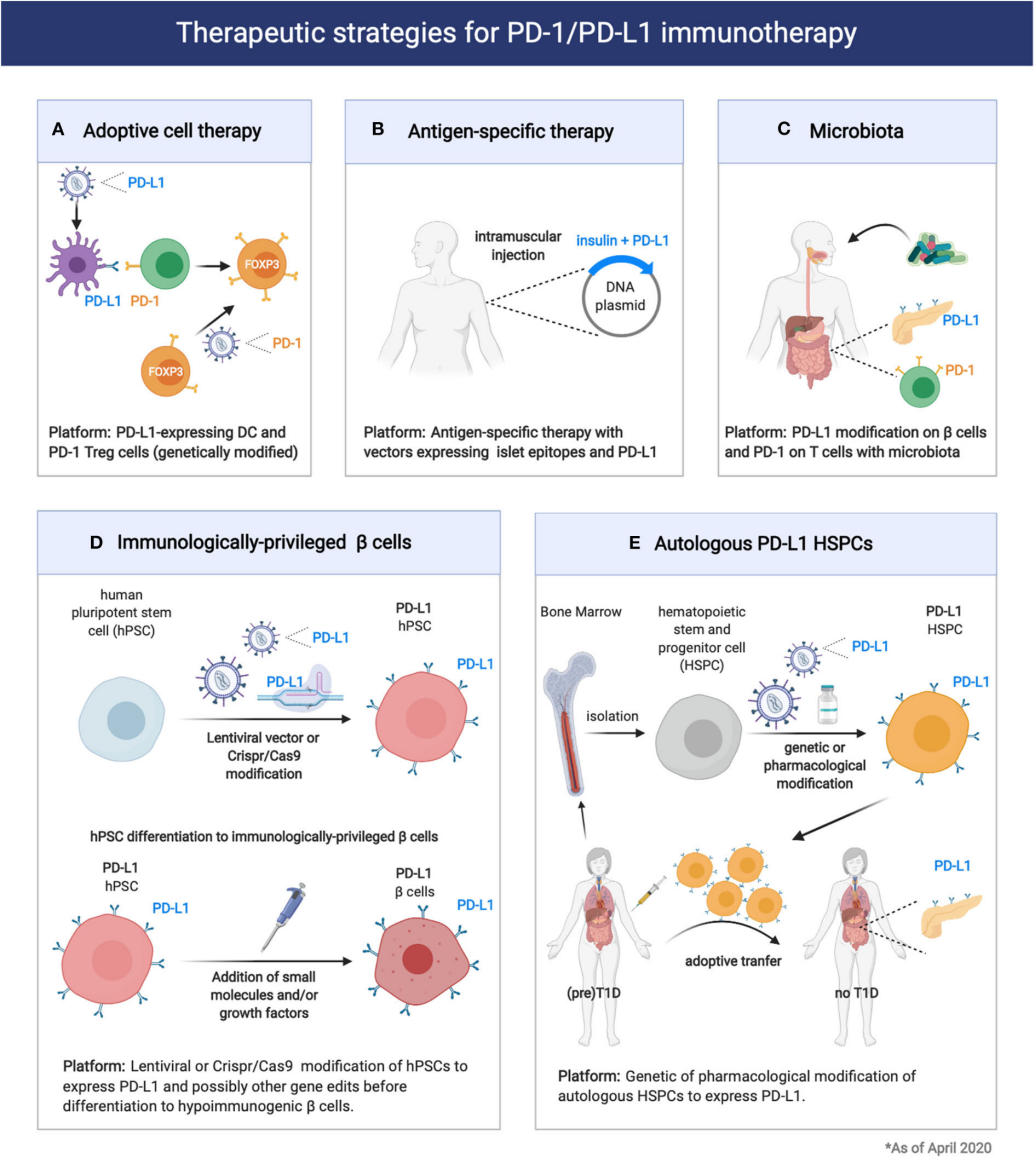
Therapeutic strategies for exploitation of the PD1/PD-L1 pathway in prevention of Type 1 Diabetes and pancreatic islet transplantation. **(A,B)** Expression of PD-1 or PD-L1 on engineered immune cells (DC, Treg cells) and tissues could enhance immune tolerance and therapeutic efficacy of adoptive cell therapy or antigen-specific therapy in T1D. **(C)** Modification of the microbiota composition through probiotic or prebiotic administration can modulate T1D pathogenesis acting on the PD1/PD-L1 axis. **(D,E)** Induction of PD-L1 expression on engineered 13 cells or HSPCs could enhance transplantation tolerance in T1D patients.

Considering the important role of the PD-1/PD-L1 pathway in controlling β cell autoimmunity and in maintaining immune homeostasis in pancreatic tissues is possible to envision several therapeutic approaches that target this inhibitory pathway for T1D prevention and/or treatment (Figure 2). In particular, Adoptive cell therapy (ACT) with tolerogenic dendritic cells (DCs) and Tregs is explored as a promising standalone or combination therapy to counter-regulate β cell autoimmunity in T1D (95). At this moment, there is one completed (96) and one ongoing phase I clinical trial led by Dr. Roep with autologous tolerogenic DCs in patients with new onset T1D (CT No: NTR5542). Over the years, several protocols of tolerogenic DCs have been developed, with and without *in vitro* supplied antigen [reviewed here (95)]. Tolerogenic DCs are thought to act via Treg expansion and induction, T-cell deletion, T-cell anergy and hyporesponsiveness. DCs can also be genetically modified with viral vectors to acquire stable immunogenic or tolerogenic properties (97). Li et al., reported genetically modified DCs expressing T-cell co-inhibitory receptor BTLA that induced CD8 T-cell tolerance and decreased diabetes in NOD mice (98). More recently, Gudi et al., showed that DCs can be efficiently engineered to simultaneously express multiple T cell repressor receptor-selective ligands (among them PD-L1) using a lentiviral transduction approach. These engineered DCs induced profound inhibition of T cell proliferation, modulation of cytokine response, and Treg cell induction, and prevented experimental autoimmune thyroiditis (99). Thus, DCs could be genetically modified to express PD-L1 (Figure 2). These DCs could in turn give rise to more Treg cells that express elevated levels of PD-1. Alternatively, Treg cells can be genetically modified to express PD-1 together with a desired, preferably islet-specific, antigen-specificity (100) (Figure 2). Further preclinical development and research will be required to address the effectiveness of PD-L1-expressing DC and PD-1-expressing Treg for the treatment of T1D. These approaches could be exploited to preserve residual β cell mass in newly diagnosed T1D patients (stage 3 T1D) as well as to prevent occurrence of clinical T1D in autoantibodies positive individuals (stage 1/2 T1D).

The antigen-specific response that characterizes T1D has been extensively studied and remains a “hot” area of investigation. Thus far, the primary antigenic drivers of the autoimmune damage are antigens which are expressed exclusively in the β cells. Proinsulin is the major antigen of the immune response and often the first adaptive immune response to be detected (insulin autoantibodies) (101, 102). Other β cell antigens are also targeted including preproinsulin (PPI), glutamic acid decarboxylase (GAD), tyrosine phosphatase-like insulinoma antigen (IA2, also called ICA512), zinc transporter ZnT8, and islet-specific glucose-6-phosphatase catalytic subunit-related protein (IGRP) (103). Antigen-specific therapies are tolerogenic approaches that rely on the administration of islet antigens as peptides, whole proteins or DNA vaccines (36, 43, 104). Antigen-specific therapies in general have not been successful so far in inducing durable protection from T1D in autoantibodies positive individuals (stage 1/2 T1D), and current studies focus on potentiating the tolerogenic outcome with combination or more than one epitopes or combination of epitopes with other tolerogenic molecules (105). DNA-based antigen-specific therapies present several unique advantages, as DNA vectors (plasmids) are easy and cheap to be produced and can guarantee prolonged expression of the encoded antigens and co-expressing factors. A possible tolerogenic approach would include the incorporation in the same DNA vector proinsulin or the major epitopes from multiple β-cell antigens and PD-L1 (Figure 2). This approach, although not yet tested, might prove more effective at delaying T1D in individuals with autoantibodies positivity (stage 1/2 T1D) but also genetically ‘‘at risk'' individuals.

Another way to therapeutically exploit the PD-1/PD-L1 axis for T1D prevention in genetically “at risk” and autoantibodies positive individuals would be through the modification of the microbiota. As we previously mentioned, a key role for the microbiota in controlling the PD-1/PD-L1 pathway expression and function was recently identified (85). Thus, modulation of the microbiota composition through administration of probiotics or dietary approaches could enhance PD-L1 expression on pancreatic β cells as well as PD-1 on Treg cells (Figure 2).

So far, we discussed how the PD-1/PD-L1 axis could be exploited to control β cell autoimmunity and preserve β cell mass in individuals with newly diagnosed T1D (stage 3 T1D) or with high genetic risk to develop disease with or without autoantibodies positivity (stage 0 and stage 1/2 T1D). For T1D patients with established disease (stage 4 T1D) pancreatic islet grafts is the main therapeutic option to restore insulin independence. Importantly, the PD-1/PDL1 pathway can also be exploited to enhance immune tolerance and promote survival of transplanted islets. The most significant limitations of clinical islet transplantation include the paucity of pancreas organ donors and the adverse effects of chronic immunosuppression. Thus, significant effort is devoted to the generation of a replenishable supply of insulin-producing cells, such as porcine pancreatic islets (106) or β cells derived from stem cells (107). Regardless of the β cell source, immunomodulatory approaches that control alloreactivity and the recurrence of autoimmunity are required. The PD-1 pathway seems to regulate autoreactive, as we previously discussed, but also alloreactive immune responses. PD-L1 blockade was shown to enhance alloreactive T cell responses and accelerated MHC class II–mismatched skin graft rejection in mice (108). A dimeric form of PD-L1 and Ig fusion protein (PD-L1.Ig) in combination with anti-CD154 blockade prevented cardiac, corneal and pancreatic islet allograft rejection, providing direct evidence for the potential of this pathway to induce allograft tolerance (109–111). Recently, a recombinant form of PD-L1 chimeric with core streptavidin (SA) (SA-PD-L1) engineered islets approach was evaluated in a preclinical model of allogeneic islet transplantation (112). SA-PDL1–engineered islets survived indefinitely in allogeneic hosts under a short course of rapamycin regimen, demonstrating the significant potential of PD-1 pathway for modulating alloreactive responses to overcome graft rejection.

Given the importance of the PD-1/PD-L1 pathway in islet-specific T cell tolerance, some investigators are using techniques of genetic engineering to generate β cells that would be immunologically “privileged” to be used for β cell replacement in T1D patients (113, 114). There are several efforts placed for generating a replenishable supply of hypoimmunogenic β cells from human pluripotent stem cells (hPSCs) using state-of the-art genome editing technologies (115). Among the multiple key genome edits that are being tried, elimination of HLA Class I and II as well as inducible overexpression of CTLA4Ig and PD-L1 are included (Figure 2). The successful generation of functional, immunologically “privileged” β cells would pave the way for a “universal off-the-shelf” transplantation platform avoiding the risks of immunosuppression and/or encapsulation and could be a “game-changer” in the race to cure T1D (5, 16).

Recently, normoglycemia in patients with recently diagnosed T1D (stage 3 T1D) was obtained with the Voltarelli trial (35, 116, 117). In this trial (117), autologous hematopoietic stem and progenitor cell (HSPC) transplantation in combination with thymoglobulin plus cyclophosphamide as induction therapy in 65 patients with newly diagnosed T1D showed to achieve insulin independence in nearly 60% of treated patients (117). This important finding suggested that HSPCs may be a therapeutic option for new onset T1D patients. Interestingly, the immunoregulatory properties of HSPCs in T1D appear to be linked to the expression of the immune checkpoint PD-L1. Recently, Ben Nasr et al., evaluated the levels of PD-L1 expression in HSPCs in both NOD mice and T1D patients (118). By means of transcriptomic profiling, flow cytometric analysis, RT-PCR, and direct analysis of bone marrow, they found a defect in the expression of PD-L1 expression in HSPCs in both NOD mice and T1D patients. To overcome the PD-L1 defect, they developed genetic (generation of PD-L1.Tg HSPC) and pharmacological approaches (treatment with IFN-β, IFN-γ, and polyinosinic-polycytidylic acid [poly(I:C)]), which successfully abrogated the autoimmune response in NOD mice (118). Tracking studies suggested that PD-L1.Tg HSPCs preferentially homed to the inflamed pancreas (119). Pharmacologically modulated HSPCs also markedly abrogated CD4- and CD8-restricted autoimmune responses and reverted diabetes in nearly 40% of newly hyperglycemic NOD mice (118). Thus, PD-L1-expressing HSPCs hold great promise for the treatment of T1D in humans (Figure 2).

## Summary

The PD1/PD-L1 dyad is very important to maintain immune homeostasis and to promote tolerance in peripheral tissues. Recent evidence indicates that the PD1/PD-L1 pathway is fundamental to prevent autoimmune diabetes so that, in some patients undergoing treatment with ICI, blocking this inhibitory pathway is sufficient to unleash islet-reactive T cells and trigger T1D. The PD1-PD-L1 axis could affect islet autoimmunity through different mechanisms involving innate and adaptive immune cells and taking place in draining lymph nodes as well as in the pancreatic tissue. The important therapeutic implication of those findings is that restoring the PD-1/PD-L1 function could represent a valid strategy to treat T1D at different stages: to counter-regulate β cell autoimmunity and prevent T1D in individuals genetically at-risk or autoantibodies positive (Stage 1/2), to promote immune tolerance and preserve residual β cell mass in new onset T1D patients (Stage 3) and, finally, to reduce alloreactive responses and favor survival of transplanted islets in T1D patients with established disease (Stage 4). Targeting the PD-1/PD-L1 has been already proven as an effective approach to promote immune tolerance in T1D and islet transplantation. Additional knowledge about the factors that regulate the PD1/PD-L1 pathway will pave the way to more effective treatments for T1D and islet transplantation.

## Author Contributions

MF reviewed the literature and wrote the manuscript. GF reviewed the literature, wrote the manuscript, and prepared the figures. All authors contributed to the article and approved the submitted version.

## Conflict of Interest

The authors declare that the research was conducted in the absence of any commercial or financial relationships that could be construed as a potential conflict of interest.
